# Transcatheter Aortic Valve Replacement in a Young Patient With Mandibuloacral Dysplasia

**DOI:** 10.1016/j.jaccas.2021.04.021

**Published:** 2021-06-16

**Authors:** Ada C. Stefanescu Schmidt, Edward T. Carreras, Marie D. Gerhard-Herman, Tsuyoshi Kaneko, Anne Marie Valente, Pinak B. Shah

**Affiliations:** a Cardiovascular Division, Department of Medicine, Brigham and Women’s Hospital and Harvard Medical School, Boston, Massachusetts, USA; b Division of Cardiac Surgery, Brigham and Women’s Hospital and Harvard Medical School, Boston, Massachusetts, USA; c Department of Cardiology, Boston Children’s Hospital, Boston, Massachusetts, USA

**Keywords:** aortic valve stenosis, mandibuloacral dysplasia, progeria, transcatheter aortic valve replacement, TAVR, transcatheter aortic valve replacement

## Abstract

A young woman with mandibuloacral dysplasia, a syndrome on the progeria spectrum with accelerated vascular calcification and calcific valve stenosis, presented with symptomatic severe aortic stenosis. She underwent transcatheter aortic valve replacement with a balloon-expandable valve, and her exertional symptoms improved significantly. (**Level of Difficulty: Intermediate.**)

## Central Illustration

**Figure:**
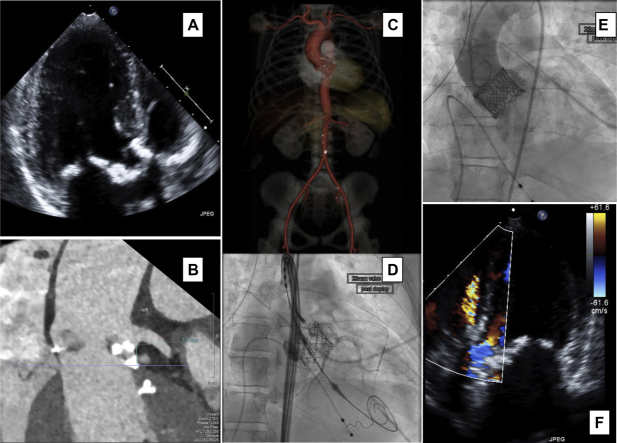

A 29-year-old woman with calcific aortic stenosis presented with exertional dyspnea and angina. She had been diagnosed with mandibuloacral dysplasia (type A on the basis of phenotype, mutation not known), a very rare autosomal-recessive premature aging syndrome including short stature, mandibular and clavicular hypoplasia, and lipodystrophy, caused by mutations in lamin A/C or zinc metalloproteinase genes (1); accelerated vascular and valvular calcifications have been described.

**Figure 1:**
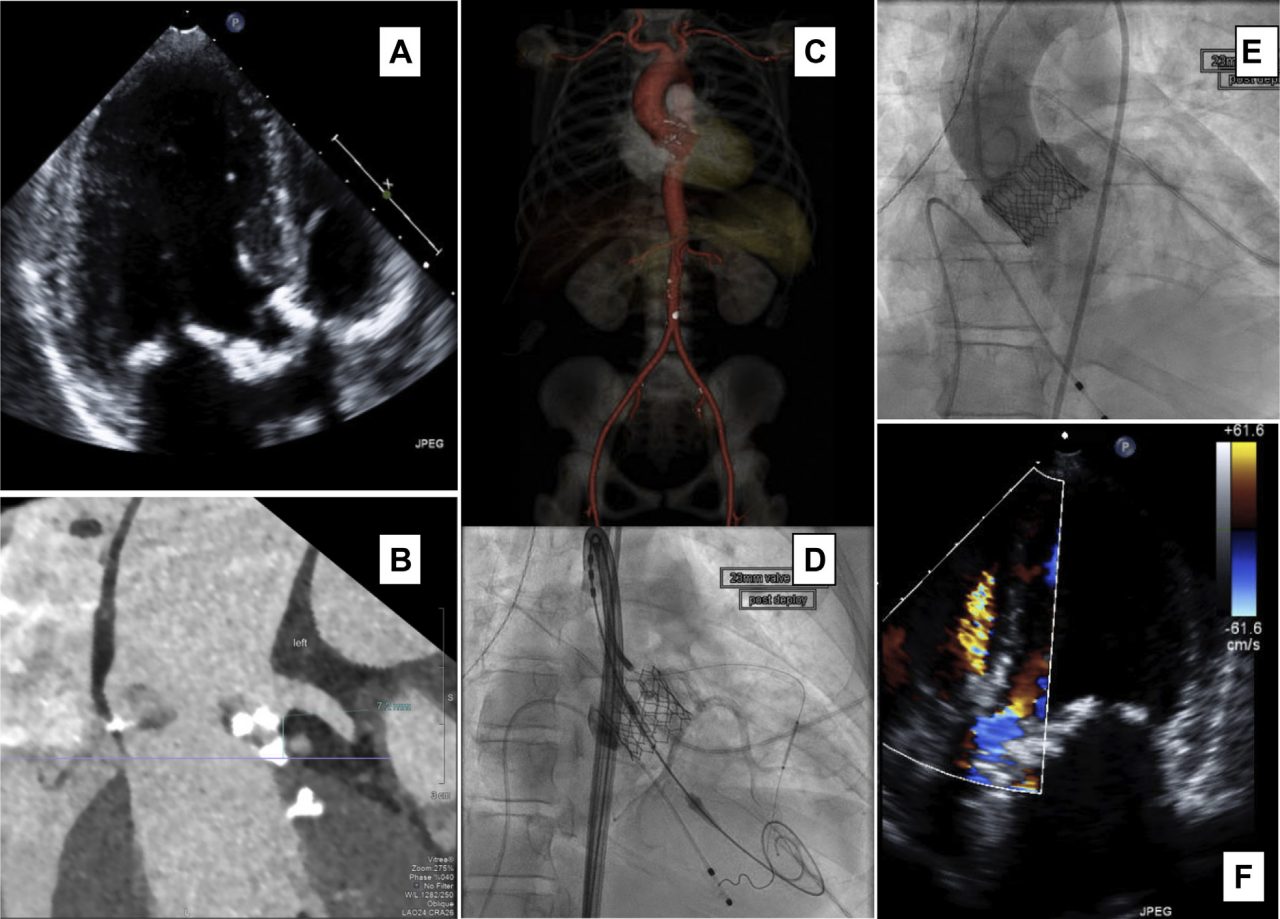
Multimodality Imaging Used for Procedural Planning **(A)** Calcification of the mitral and aortic valves on echocardiography. **(B)** On gated cardiac computed tomography, short left main coronary ostial height and calcific nodule in the left ventricular outflow tract were appreciated. **(C)** Computed tomographic reconstruction of aorta and iliofemoral vessels. **(D)** Nonselective left coronary injection after valve deployment showing patent left main coronary artery and undeployed coronary stent in the left anterior descending coronary artery. **(E)** Final aortic root angiography. **(F)** Post-procedural echocardiography, with normal gradients and no significant paravalvular leak but suggestion of a fistula between the aorta and right ventricular outflow tract.

The patient had a heavily calcified trileaflet aortic valve, with peak antegrade velocity of 5.2 m/s, a mean gradient of 64 mm Hg, and moderate calcific mitral stenosis. She weighed 33 kg and had lipodystrophy but no metabolic syndrome. Computed tomographic angiography showed only minimally calcified but small iliofemoral vessels (Figure 1). Her surgical risk, assessed by 1 adult and 1 pediatric cardiac surgeon, was underestimated by the Society of Thoracic Surgeons risk score (0.9%), which does not adjust for her small body size (below cutoff), chest wall geometry, and tissue fragility (Supplemental Table 1); the patient prioritized a rapid recovery. After multidisciplinary evaluation and heart team meeting, she was offered transcatheter aortic valve replacement (TAVR), guided by pre-procedural echocardiography and cardiac computed tomography (Figure 1), with specific discussion of risks (Supplemental Table 1).

Access was obtained under ultrasound and fluoroscopic guidance, and the delivery sheath was inserted without complications. After balloon valvuloplasty, a 23-mm SAPIEN S3 valve (Edwards Lifesciences, Irvine, California) was placed under rapid pacing (Videos 1 and 2). Immediate post-procedural echocardiography and aortic angiography (Video 3) showed a well-seated valve with only trace paravalvular regurgitation and unobstructed flow in the coronary arteries, so the undeployed coronary stent that was prophylactically placed in the left anterior descending coronary artery was removed. She was hemodynamically stable and was extubated in the procedure room. On day 2, echocardiography revealed a small aortic-to-right ventricle fistula (Video 4), likely caused by a perforation of the membranous ventricular septum from a calcific nodule at the base of the right coronary cusp. Her dyspnea on exertion and angina resolved completely. One year later, she remained asymptomatic, with good cardiovascular fitness. The prosthetic valve showed normal hemodynamic status, and the small aortic–to–right ventricle fistula remained stable with no right ventricular enlargement.

To our knowledge, this is only the second reported case of TAVR in a patient with mandibuloacral dysplasia (2). Small body size, valvular calcifications, and vascular fragility are important to take into account when planning any procedure. Aortic perforation is a rare complication of TAVR (<1%), in particular in patients with heavily calcified valves and small annuli (3). Perforation into a cardiac chamber (most commonly the right ventricle or right atrium) can often be managed conservatively, as long as the shunt is not hemodynamically significant and there is no hemolysis. In this case, the bulky valve calcification, small sinus diameters, and tissue fragility contributed to this complication.

## Funding Support and Author Disclosures

Dr. Kaneko is a consultant for Edwards Lifesciences and Medtronic; and is a speaker for Abbott. Dr. Shah is a proctor for Edwards Lifesciences; and has received educational grants from Edwards Lifesciences, Medtronic, and Abbott.

## Appendix

Supplemental Video 1 Initial Aortogram Showing Calcific Aortic Stenosis and Small Aortic Root

Supplemental Video 2 Deployment of Balloon-Expandable Valve Under Rapid Ventricular Pacing

Supplemental Video 3 Final Aortogram Showing Well-Positioned Balloon-Expandable Valve, With Trace Paravalvular Leak and Trace Aortic-to-Right Ventricular Fistula

Supplemental Video 4 Post-Procedural Apical 5-Chamber View on Echocardiography, Showing a Small High-Velocity Systolic and Diastolic Jet Between the Right Coronary Cusp and Right Ventricle
