# Supplementary material for: Transcatheter Aortic Valve Replacement in a Young Patient With Mandibuloacral Dysplasia
Source: JACC Case Rep. 2021 Jun 16;3(6):897–9. doi: 10.1016/j.jaccas.2021.04.021 (PMC8311272; doi:10.1016/j.jaccas.2021.04.021)
Supplement: Supplemental Table 1 [file mmc5.docx]

**Supplemental Materials for “Transcatheter aortic valve replacement in a young patient with mandibuloacral dysplasia”**

Ada C. Stefanescu Schmidt MD MSc^a^, Edward T. Carreras MD^a^, Marie D. Gerhard-Herman MD^a^, Tsuyoshi Kaneko MD^b^, Anne Marie Valente MD^a,c^, Pinak B. Shah MD^a^

**Supplemental Table 1: Anatomic risk factors and management for patients with mandibuloacral dysplasia, a premature aging syndrome associated with lamin A/C mutations**

|  | Risk factors for surgical approach | Risk factors for transcatheter procedure | Management during TAVR |
| --- | --- | --- | --- |
| Mandibuloacral dysplasia associated tissue fragility | Concern about dehiscence of cardiac tissue  Increased risk of bleeding  Poor wound healing  Risk of delayed chest closure  Longer recovery period  Difficult peripheral venous access | Concern about dissection of aorta and ileofemoral vessels  Difficult peripheral venous access | Ultrasound, fluoroscopy-guided access |
| Anesthesia considerations | High risk airway, difficult intubation given mandibular dysplasia, micrognathia, small oral opening, narrowed nose, decreased flexibility of the neck and temporo-mandibular joints  Increased risk of hypothermia given decreased subcutaneous fat | High risk airway | Pre-operative anesthesia consult, pediatric-sized equipment and fiberoptic intubation recommended |
| Calcified annulus | Risk of stroke  Risk of annular injury  Risk of small valve implantation | Risk of paravalvular leak, annular rupture | Appropriate valve sizing by preprocedural cardiac CT |
| Calcification in left ventricular outflow tract | Requirement for debulking | Risk of paravalvular leak and annular rupture | Appropriate sizing, avoiding low-lying implantation |
| Low-lying coronary ostia | Risk of coronary obstruction if large prosthesis is implanted | Risk of coronary obstruction from native leaflets pinned by new valve | Sizing by preprocedural cardiac CT; coronary protection by placing an undeployed coronary stent in the coronary artery (left anterior descending in this case) prior to balloon valvuloplasty; the coronary guide catheter is withdrawn to the ascending aorta during valve deployment and ready to re-engage coronary and deploy stent in the ostium of the coronary if it is obstructed after valve implantation |
| Small diameter of ileofemoral arteries | Difficult access if intraaortic balloon pump is required | Risk of dissection during sheath entry  Risk of vascular injury | Sizing by pre-procedural CT; lower risk if less calcifications. Angiogram through the sheath prior to removal to assess for vessel injury which can be treated percutaneously prior to sheath removal |
